# Predictive signature of static and dynamic functional connectivity for ECT clinical outcomes

**DOI:** 10.3389/fphar.2023.1102413

**Published:** 2023-01-23

**Authors:** Zening Fu, Christopher C. Abbott, Jing Sui, Vince D. Calhoun

**Affiliations:** ^1^ Tri-Institutional Center for Translational Research in Neuroimaging and Data Science (TReNDS), Georgia Institute of Technology, Georgia State University, Emory University, Atlanta, GA, United States; ^2^ Department of Psychiatry, University of New Mexico, Albuquerque, NM, United States; ^3^ State Key Laboratory of Cognitive Neuroscience and Learning, Beijing Normal University, Beijing, China; ^4^ Department of Electrical and Computer Engineering, Georgia Institute of Technology, Atlanta, GA, United States

**Keywords:** resting-state functional MRI, dynamic functional connectivity, ECT outcomes prediction, antidepressant outcomes, cognitive changes

## Abstract

**Introduction:** Electroconvulsive therapy (ECT) remains one of the most effective approaches for treatment-resistant depressive episodes, despite the potential cognitive impairment associated with this treatment. As a potent stimulator of neuroplasticity, ECT might normalize aberrant depression-related brain function via the brain’s reconstruction by forming new neural connections. Multiple lines of evidence have demonstrated that functional connectivity (FC) changes are reliable indicators of antidepressant efficacy and cognitive changes from static and dynamic perspectives. However, no previous studies have directly ascertained whether and how different aspects of FC provide complementary information in terms of neuroimaging-based prediction of clinical outcomes.

**Methods:** In this study, we implemented a fully automated independent component analysis framework to an ECT dataset with subjects (n = 50, age = 65.54 ± 8.92) randomized to three treatment amplitudes (600, 700, or 800 milliamperes [mA]). We extracted the static functional network connectivity (sFNC) and dynamic FNC (dFNC) features and employed a partial least square regression to build predictive models for antidepressant outcomes and cognitive changes.

**Results:** We found that both antidepressant outcomes and memory changes can be robustly predicted by the changes in sFNC (permutation test p < 5.0 × 10^−3^). More interestingly, by adding dFNC information, the model achieved higher accuracy for predicting changes in the Hamilton Depression Rating Scale 24-item (HDRS_24_, t = 9.6434, p = 1.5 × 10^−21^). The predictive maps of clinical outcomes show a weakly negative correlation, indicating that the ECT-induced antidepressant outcomes and cognitive changes might be associated with different functional brain neuroplasticity.

**Discussion:** The overall results reveal that dynamic FC is not redundant but reflects mechanisms of ECT that cannot be captured by its static counterpart, especially for the prediction of antidepressant efficacy. Tracking the predictive signatures of static and dynamic FC will help maximize antidepressant outcomes and cognitive safety with individualized ECT dosing.

## Introduction

Characterized by depressed mood, abnormal psychomotor activity, and impaired cognitive function, major depressive disorder (MDD) is amongst the most prevalent debilitating diseases worldwide (Kessler et al., 2003), affecting approximately 300 million individuals in 2017 (James et al., 2018). Besides its pervasiveness, MDD also has a high recurrence rate (about 80% of patients in remittance experience at least one recurrence) (Vos et al., 2004) and shows great heterogeneity in symptoms that might influence that diagnosis and treatment (Malgaroli et al., 2021). Fortunately, there are numerous effective treatments for treatment-resistant depressive episodes, such as electroconvulsive therapy (ECT), transcranial magnetic stimulation, and other brain stimulation therapies. As an exceptionally effective medical treatment for MDD patients, ECT provides effective and safe antidepressant outcomes by applying brief electrical stimulation to the scalp to induce changes in neurotransmitter levels and improve neuroplasticity (Bahji et al., 2019).

Independent of the antidepressant outcomes, the stigma and fear of ECT-mediated cognitive impairment reduce ECT utilization (Wilkinson et al., 2021). ECT cognitive meta-analyses demonstrate acute but transient cognitive impairment immediately after the acute phase of the series (Semkovska and McLoughlin, 2010; Semkovska et al., 2011). Numerous mechanisms of ECT cognitive impairment have been proposed, including excessive neuroplasticity and disrupted long-term potentiation (Ousdal et al., 2022). However, the findings on ECT-induced changes in cognition are somehow contradictory, with substantial cognitive deficits (Sackeim et al., 2007; Semkovska and McLoughlin, 2010) and improvement in cognitive function (Bosboom and Deijen, 2006; Fujita et al., 2006) documented in the literature. Due to this inconsistency, the precise characterization of cognitive changes is still a debated topic in this area. ECT-mediated functional brain neuroplasticity has been implicated in antidepressant and cognitive outcomes (Wang et al., 2020; Fu et al., 2021c). Functional connectivity (FC) changes show relationships with ECT-induced symptom improvements and cognitive changes (Perrin et al., 2012; Wang et al., 2020), where different brain regions can have diverse associations with clinical outcomes. For instance, while left hippocampal FC did not change significantly, right hippocampal FC increased after ECT treatment, correlating with depressive symptom reduction (Abbott et al., 2014). Such regional heterogeneity of FC to ECT outcomes, when considered in isolation, poses a challenge in precisely characterizing brain signatures of ECT responses. Existing studies have used the machine learning tool to combine a set of distributed FC features into a single index for predicting ECT-induced outcomes (Moreno-Ortega et al., 2019; Long et al., 2020; Yang et al., 2020). Potentially most exciting is the demonstration that combining different resting-state brain FC can increase the predictive power for the ECT treatment responses (Moreno-Ortega et al., 2019).

Despite such progress, the previous study using FC for ECT outcome prediction has been limited by an implicit assumption of spatial and temporal stationarity in the relationships between brain regions. The human brain is a highly dynamic system with continuously changing local activation and inter-communication even during the resting-state (Gray, 1990; Berger, 2011; Allen et al., 2014). The temporal variation in FC reflects the brain adaptation to both internal and external stimuli (Chang and Glover, 2010; Allen et al., 2018), which might represent functional neuroplasticity, a key brain mechanism associated with ECT. Recent studies have found prominent relationships between dynamic FC changes and antidepressant outcomes and clinical changes after ECT (Fu et al., 2021c; Dini et al., 2021). However, some challenging issues related to FC-based prediction of ECT outcomes remained unaddressed. First, previous studies focused on static FC and dynamic FC in isolation and did not combine them into the prediction model. The additional predictive potential of dynamic FC to static FC is unknown. Second, the utility of FC-predicted clinical outcomes is generally established by probing the predictive signatures for the antidepressant efficacy and cognitive deficits alone. Previous investigations have yet to disentangle distinct from potential overlapping antidepressant and cognitive FC patterns in predictive modeling.

In this work, we used an FC-based machine learning approach within full cross-validation (CV) analyses to probe reliable and robust imaging signatures for the ECT-induced antidepressant outcomes and cognitive changes from whole-brain static and dynamic FC. We hypothesized that the dynamic information in FC is not redundant but conveys additional mechanisms of ECT to the static FC analysis. Specifically, we hypothesized that 1) both antidepressant outcomes and cognitive changes can be reliably predicted by unique patterns of static FC; and 2) adding dynamic FC information improves accuracy for predicting antidepressant outcomes or cognitive changes relative to static FC alone.

## Materials and methods

### Study design and participants

The present study used an ECT dataset approved by the University of New Mexico Human Research Protections Office (Abbott et al., 2021). Participants signed procedural consent or assented to the investigation under protocols approved by the Institutional Review Board. 62 participants aged between 50 and 80 years old were recruited from December 2016 to September 2019, who had a diagnosis of major depressive disorder and met the clinical indication for ECT. This investigation focused on mid-life and older patients (age range: 50–80 years) with major depressive disorder. Clinically, this age range is the optimal laboratory to investigate targeted brain engagement and clinical outcomes (Abbott et al., 2021). Computer modeling has demonstrated the importance of age-related brain changes in relation to ECT-induced electric field strength (Deng et al., 2015). In addition, lateralized brain neuroplasticity is most evident in older patients treated with right unilateral electrode placement (Abbott et al., 2014; Dukart et al., 2014). Older age is also associated with an increased probability of response (Tew et al., 1999) and ECT-mediated cognitive impairment (Squire and Chace, 1996). The risk of cognitive impairment, which is both scientific (Sterling, 2000; Frank, 2002) and public (Breggin, 2008) opponents of ECT as evidence of brain damage, deters the patient or the surrogate decision maker from this potentially life-saving treatment. The reversal of disease-related regional brain connectivity in this older patient sample is significant because it will demonstrate neuroplasticity throughout the life span as a therapeutic antidepressant mechanism. All participants tapered and discontinued their psychotropic medications before the assessment to minimize the medication confounds. Details of the inclusion and exclusion criteria can be found in (Abbott et al., 2021).

Participants were randomly assigned to three pulse amplitudes [600, 700, or 800 milliamperes (mA)] (Sackeim et al., 2000; Abbott et al., 2021), starting the ECT treatment with right unilateral electrode placement with either an ultra-brief pulse width [0.3 milliseconds (ms)] or brief pulse width (1.0 ms). Raters and subjects were blinded to randomization. Participants received clinical and cognitive assessments and the imaging session before ECT (v1), after the first six ECT treatments (v2), and within 1 week of finishing all ECT series (v3). The primary clinical outcomes are depression severity measured by the Hamilton Depression Rating Scale 24-item (HDRS_24_) total score and cognition measured by Hopkins Verbal Learning Test (HVLT)-total Repetition (HVLT-R) and Delayed Recall (HVLT-DR) scores. If subjects had a <25% reduction in HDRS_24_ at v2 (compared to the assessment at v1), they were switched to bitemporal electrode placement with a brief pulse for the remaining ECT series. We have uploaded the ECT imaging (unprocessed), clinical, and demographic data to the National Data Archive. More details of the study design, participants, and clinical and cognitive assessments can be found in Abbott et al. (2021).

### Image acquisition and preprocessing

Resting-state functional magnetic imaging (fMRI) data were collected by a 3T-Siemens scanner with the following parameters: repetition time (TR) = 745 ms, echo time = 29 ms, flip angle = 75°, slices = 192, voxel size = 2.0 × 2.0 × 2.0 millimeter (mm)^3^, and total acquisition time 4:58 (minutes:seconds). Participants received one or two resting-state scans at each visit. We preprocessed the resting-state fMRI data *via* a combination of the FMRIB Software Library v6.0 toolbox and the Statistical Parametric Mapping 12 toolbox, under the MATLAB 2020b environment. Specifically, we performed slice timing correction followed by distortion correction. Then we corrected the head motion and normalized the data to the standard Montreal Neurological Institute (MNI) space. Finally, we smoothed the fMRI data with a 6 mm Gaussian kernel.

We performed quality control (QC) on the preprocessed fMRI data for selecting subjects for further analysis. Subjects were excluded if their head motions were larger than 3 mm translations or 3° rotations. We also excluded subjects if their images did not show good normalization to the MNI standard space (by comparing the individual mask with the group mask). Details of the preprocessing and QC criteria are provided in the Supplementary Material.

### Analysis flowchart

**FIGURE 1:**
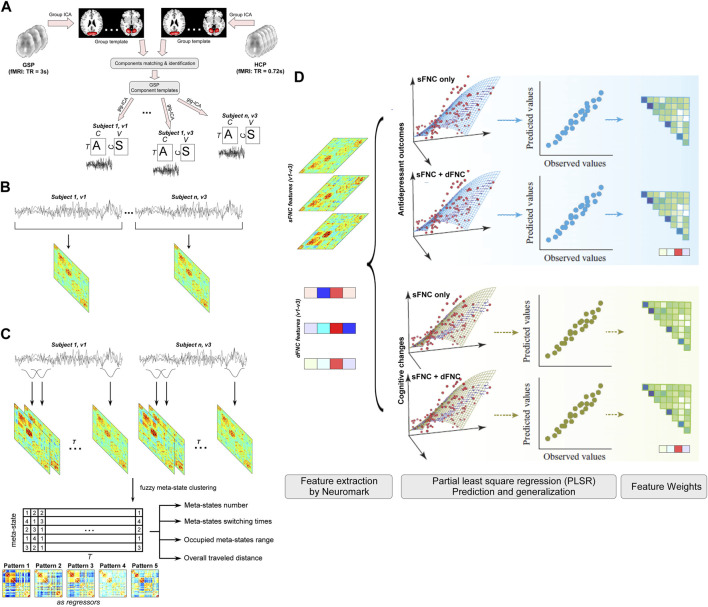
Flowchart of the prediction analyses. To examine whether ECT-induced antidepressant outcomes and cognitive changes have neural representations on static and dynamic functional connectivity, we develop separate predictive models for HDRS and HVLT based on the sFNC features and global dynamism features and examine their weight maps at connection and functional domain levels. **(A)** Templates construction and individual network estimation. **(B)** Static functional network connectivity (sFNC) features. **(C)** Dynamic functional network connectivity (dFNC) features. **(D)** Prediction of ECT outcomes.

In this study, we build a machine learning model within fully CV analyses based on the static and dynamic connectivity features to investigate whether 1) functional network connectivity (FNC) features can predict ECT-induced depressive symptoms’ changes and cognitive changes, and 2) dynamic features provide additional information to the predictive model to enhance the accuracy. The whole analysis framework is displayed in Figure 1. Crucially, the investigation of the predictive brain signatures from both static and dynamic perspectives allows us to understand whether and how dynamic FNC (dFNC) signatures add ECT-related information to the static FNC (sFNC) features.

### Neuromark framework for extracting subject-specific networks

In this study, we applied an automated independent component analysis (ICA)-based framework, namely Neuromark (Du et al., 2020), to estimate the functional networks and the corresponding time courses (TCs) for each subject. This Neuromark framework used two large publicly available datasets to construct a group of robust network templates. The network templates were then used as the reference in the spatial-constrained ICA to extract subject-specific networks and their TCs. The identified meaningful networks were defined as intrinsic connectivity networks (ICNs). ICNs had their activation peaks located across the whole-brain gray matter areas and were labeled according to their prior anatomical and functional knowledge. Neuromark provides comparability of networks across subjects and sessions while allowing single-scan variability in the network maps. Previous studies have shown the efficacy of the Neuromark framework in capturing robust neuroimaging biomarkers in a wide range of brain diseases (Fu et al., 2020; Tu et al., 2020; Fu et al., 2021a; 2021b). We provided more details of the Neuromark framework in the Supplementary Material.

### Static functional network connectivity

After having the ICNs and TCs for each subject, we performed the post-processing steps on the TCs to remove the additional confounding effects. We first detrended linear, quadratic, and cubic trends from the TCs. Then a multiple regression was performed to regress the six realignment parameters and their derivatives. Thirdly, we removed the detected outliers from the TCs. Finally, we performed band-pass filtering with a cutoff frequency of 0.01–0.15 Hz on the TCs. Pearson correlation coefficient was calculated between the post-processed TCs to measure the sFNC. The whole-brain sFNC (C × C, C is the number of ICNs) was further z-scored to have the zero mean and standard variation.

### Dynamic functional network connectivity and global dynamism

The resting-state brain continuously integrates and coordinates different neural populations to adapt to the demands of internal and external environments. Here, we employed a sliding-window approach on the post-processed TCs to estimate the whole-brain dFNC. Within each window, a graphic lasso method was implemented to estimate the correlation matrix (C × C). We chose the window size as 20 TRs and slid the window in 1 TR, resulting in a W × C × C (W is the number of windows) array for each subject, representing the FNC changes across time.

We performed a meta-state analysis based on fuzzy k-means clustering to quantify the global dynamism of FNC (Miller et al., 2016). This analysis modeled dFNC as weighted sums of maximally independent connectivity patterns. Each dFNC was represented as a discretized vector of connectivity pattern weights, called a meta-state. Four dynamism measures were defined by the meta-state analysis, including 1) meta-states number (the number of distinct high-dimensional meta-states passed through); 2) meta-states switching times (the number of times switching among different meta-states); 3) occupied meta-states range (the divergence of the meta-states); 4) overall traveled distance (the overall traveled distance among different meta-states), reflecting high dimensional dynamic properties of the whole-brain network. The effectiveness of the meta-state analysis in capturing the overall patterns of dynamic brain networks has been proved in numerous pieces of literature (Miller et al., 2016; Mennigen et al., 2018; Fu et al., 2019).

### Development of FNC-based predictive models

Partial least square regression (PLSR) was employed to build predictive models for the percentage change of HDRS_24_ and each of the subdomains of HDRS_24_ separately. PLSR can capture reliable brain-phenotype relationships and has been widely used in predictive neuroimaging. PLSR did not require prior feature selection or prior training to achieve dimension reduction, as it worked by projecting high-dimensional features into a small set of latent components. This strength can facilitate the comparison of predictive models based on different types of features.

We placed the prediction model in a tenfold CV framework. Specifically, 90% of the data were chosen as the training set and the remaining 10% were used for the testing set. Model building was performed using another CV framework using the training set. The testing set was independent of the training process to prevent leakage between them. The predictive model, namely beta value, was directly applied to the testing set without any modifications. Considering the division of data folds was conducted randomly, we further employed shuffle-split techniques by repeating the prediction procedure 1,000 times to control this influence. Model performance was quantified as the correlation r between actual and predicted scores, averaged across 1,000 repetitions.

The sFNC changes between v1 and v3 (v1–v3) were first calculated and used as the input features in the PLSR model. Because the sFNC matrix is symmetric, we only used the upper triangle elements of the sFNC matrix, which results in 1,378 sFNC features for the predictive model. Then we added four dynamism measures, resulting in 1,382 features for the predictive model. We investigated whether adding dFNC features can improve prediction accuracy or not. We also built a PLSR model for the prediction of ECT-induced cognitive changes, which are measured by the changes in HVLT scores. A similar prediction framework was employed in building the PLSR model for predicting HVLT changes.

The relative contribution of each feature to prediction is quantified by extracting the regression coefficients from the predictive model (the returned beta in plsregress). We generated the static feature representation and the dynamic feature representation for the predictive model by averaging 10,000 weight maps (1,000 repetitions × 10 folds). To unveil how functional domains play a disproportionate role in explaining the success of predicting ECT outcomes, we grouped the whole-brain ICNs into seven functional domains according to their anatomical and functional prior information. Next, for each pair of the functional domains (between- or within-domain), weights of all FNC were added up and then normalized by the total number of FNC belonging to that pair to control for the influence of domain size.

We examined the validity and stability of predictive models through the following analyses. First, the mean and standard deviation of the sFNC and dynamic feature weights across 1000 CV were calculated and displayed. Second, to show the significance of prediction accuracy, we performed a permutation test with 1,000 iterations. Third, to confirm that the predictive models were not biased by the potential confounding effects, we regressed out the covariates of age, sex, current, treatment number, and pulse width from the input features, and reperformed the PLSR framework based on the cleaned features.

### Code availability

The codes of the Neuromark framework have been released and integrated into the group ICA Toolbox (https://trendscenter.org/software/gift/), which can be downloaded and used directly by users worldwide. Other MATLAB codes of this study can be obtained from the corresponding authors.

## Results

### Intrinsic connectivity networks

The Neuromark QC selected 50 subjects for the present study, demographic information is provided in Supplementary Table S1. The selected subjects have at least one good resting-state fMRI scan at both v1 and v3 sessions that passed the QC. The Neuromark framework identified 53 meaningful ICNs across the whole brain, which were organized into seven functional domains: subcortical (SC), auditory (AUD), visual (VS), sensorimotor (SM), cognitive-control (CC), default-mode (DM), and cerebellar domains (CB). The spatial maps of ICNs (components) were displayed in Supplementary Figure S1 and details of the ICN coordinates and labels are provided in Supplementary Table S2.

### Static and dynamic connectome-based prediction of ECT-induced antidepressant outcomes

**FIGURE 2:**
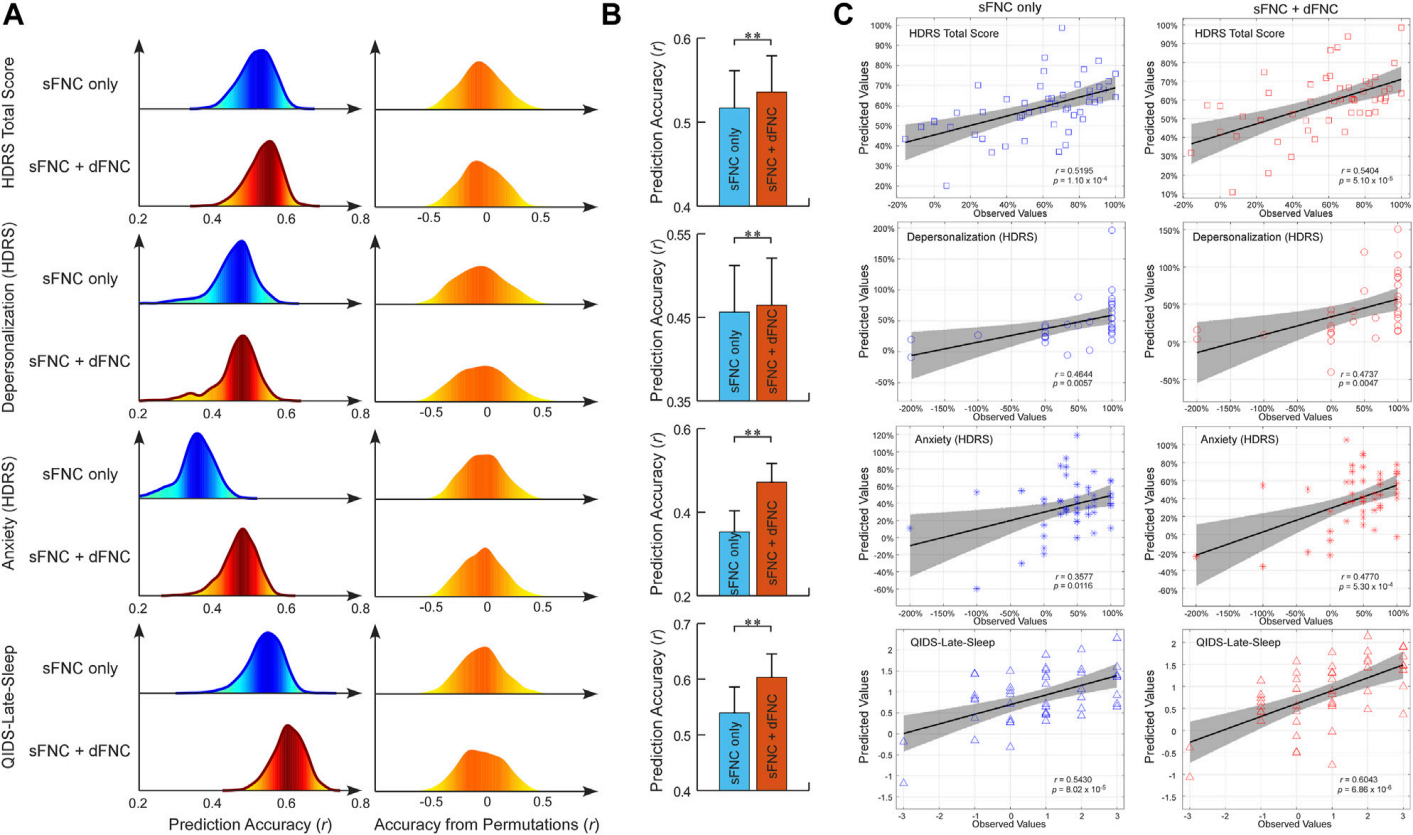
FNC-based prediction results for antidepressant outcomes induced by ECT. **(A)** Distribution of prediction accuracies across 1,000 repetitions of cross-validation and distribution of accuracies based on permutation testing across 1,000 iterations. **(B)** Statistical analysis between prediction accuracies based on sFNC features only and based on combined features (sFNC+dFNC). **(C)** Scatter plot shows prediction of changes in HDRS_24_ scores and QIDS score. Considering that the prediction framework was repeated 1,000 times, we only show results from the iteration with the median prediction accuracy.

The sFNC features were the correlations between 53 ICNs while the dFNC features were the four dynamism features captured by a fuzzy k-means clustering with five connectivity patterns. The number of connectivity patterns is within a reasonable range (4–7) in the previous studies. Based on the sFNC features, we built predictive models for antidepressant outcomes which are measured by HDRS_24_. Separate models were built for the composite HDRS_24_ and each of its sub-scores within a repeated CV framework. In Figures 2A, C, sFNC features can successfully predict the HDRS_24_ composite score changes (*r* = 0.5169 ± 0.0444, *R* ^2^ = 0.2691 ± 0.0453, permutation test *p* < 2.0 × 10^−3^). When adding dynamic features to the prediction model, the combined features can achieve better prediction accuracy (*r* = 0.5358 ± 0.0433, *R* ^2^ = 0.2889 ± 0.0455, permutation test *p* < 2.0 × 10^−3^). The two-sample t-test in Figure 2B shows that using combined features provides higher prediction accuracy compared to using only static features (*t* = 9.6434, *p* = 1.5 × 10^−21^). sFNC can also predict two sub-scores of HDRS_24_. Specifically, sFNC can predict changes in depersonalization score and anxiety score, with mean correlations between actual versus predicted values as *r* = 0.4564 ± 0.0557 and *r* = 0.3531 ± 0.0504 (permutation test *p* < 2.3 × 10^−2^). Same to the HDRS_24_ composite score, combined static features with dynamic features can increase the prediction accuracy (*r* = 0.4646 ± 0.0564, *t* = 3.2626, *p* = 0.0011, and *r* = 0.4720 ± 0.0448, *t* = 55.7600, *p* = 1.0 × 10^−30^). We also found that the sFNC features can predict the change in sleep condition which is measured by the Quick Inventory of Depressive Symptomatology (QIDS) late sleep score (*r* = 0.5394 ± 0.0466, *R* ^2^ = 0.2931 ± 0.0491, permutation test *p* < 1.0 × 10^−3^). Combining both static and dynamic FNC features can achieve better prediction accuracy (*r* = 0.6034 ± 0.0419, *R* ^2^ = 0.3658 ± 0.0502, *t* = 32.2820, *p* = 2.3 × 10^−30^). Note that, although the prediction accuracies slightly decreased after controlling for the potential covariates, they are still significantly higher than the accuracies from the permutation test (*p* < 5.0 × 10^−2^). More importantly, the models built on combined features can achieve consistently higher prediction accuracy than those built on sFNC features only (Supplementary Table S3).

### Static and dynamic connectome-based prediction of ECT-induced cognitive changes

**FIGURE 3:**
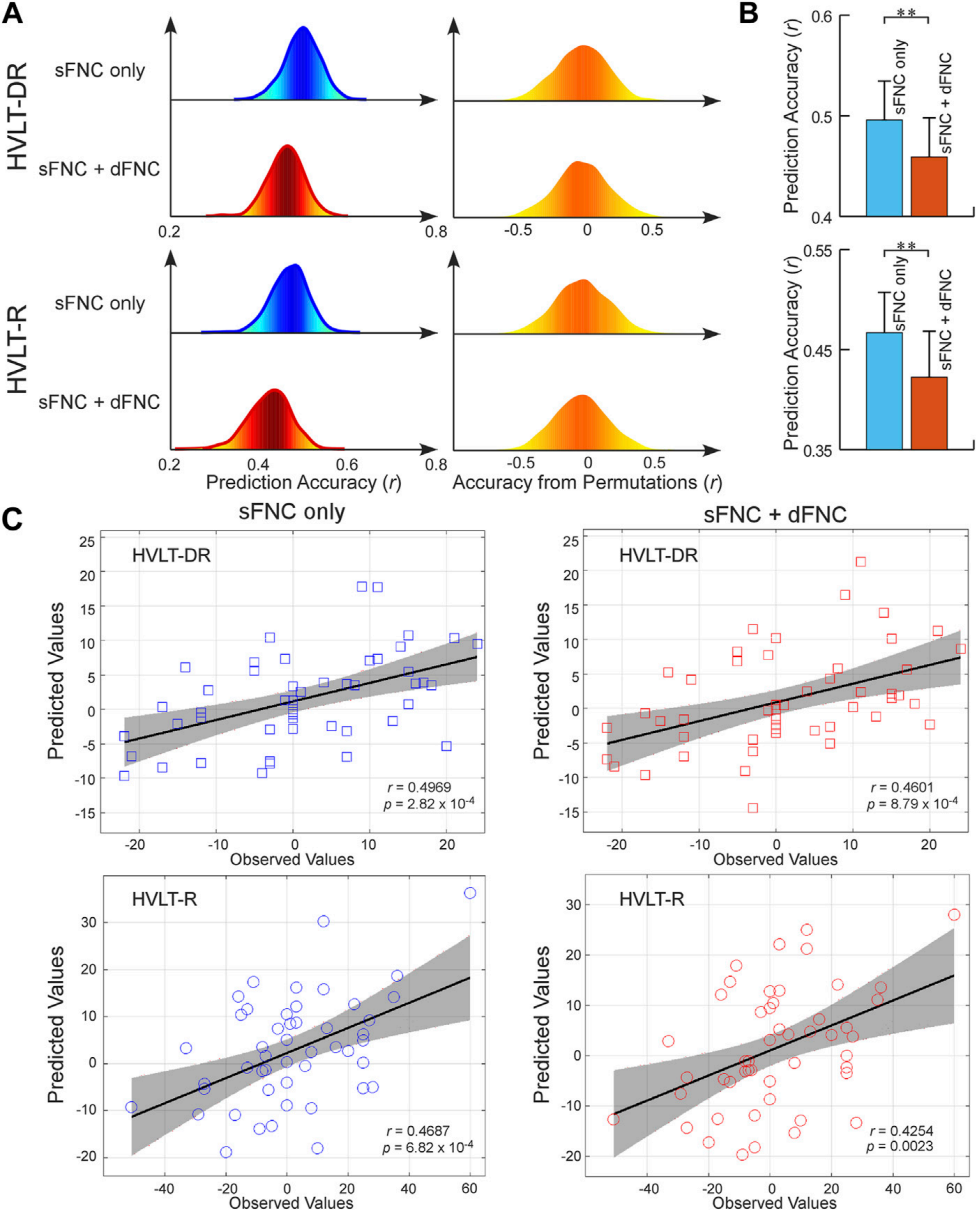
FNC-based prediction results for cognitive changes induced by ECT. **(A)** Distribution of prediction accuracies across 1,000 repetitions of cross-validation and distribution of accuracies based on permutation testing across 1,000 iterations. **(B)** Statistical analysis between prediction accuracies based on sFNC features only and based on combined features (sFNC+dFNC). **(C)** Scatter plot shows prediction of changes in HVLT scores. Considering that the prediction framework was repeated 1,000 times, we only show results from the iteration with the median prediction accuracy.

To exploit the FNC signatures for ECT-induced cognitive changes, we further built predictive models for cognitive performance measured by HVLT scores. Separate models were built for each of the HVLT t-scores within a repeated CV framework. We found that the sFNC features can predict changes in the HVLT-DR score and HVLT-R score (*r* = 0.4958 ± 0.0387, *R* ^2^ = 0.2473 ± 0.0381, and *r* = 0.4669 ± 0.0403, *R* ^2^ = 0.2196 ± 0.0374, permutation test *p* < 5.0 × 10^−3^, Figures 3A, C). Different from the prediction of antidepressant outcomes, we found that adding dFNC features to the model did not improve the prediction accuracy (*r* = 0.4590 ± 0.0388, *R* ^2^ = 0.2122 ± 0.0355, and *r* = 0.4223 ± 0.0459, *R* ^2^ = 0.1805 ± 0.0383, permutation test *p* < 1.0 × 10^−2^, Figures 3A, C). Instead, using only sFNC features provided better performance in predicting cognitive changes (*t* = −21.2111, *p* = 3.2 × 10^−20^, and *t* = −23.0381, *p* = 2.4 × 10^−30^, Figure 3B). The overall prediction results are replicated after controlling for the potential confounding effects, where models built on combined features did not improve the prediction accuracy compared to those built on sFNC features only (Supplementary Table S3).

### Predictive anatomy of intrinsic connectivity network and global dynamism

**FIGURE 4:**
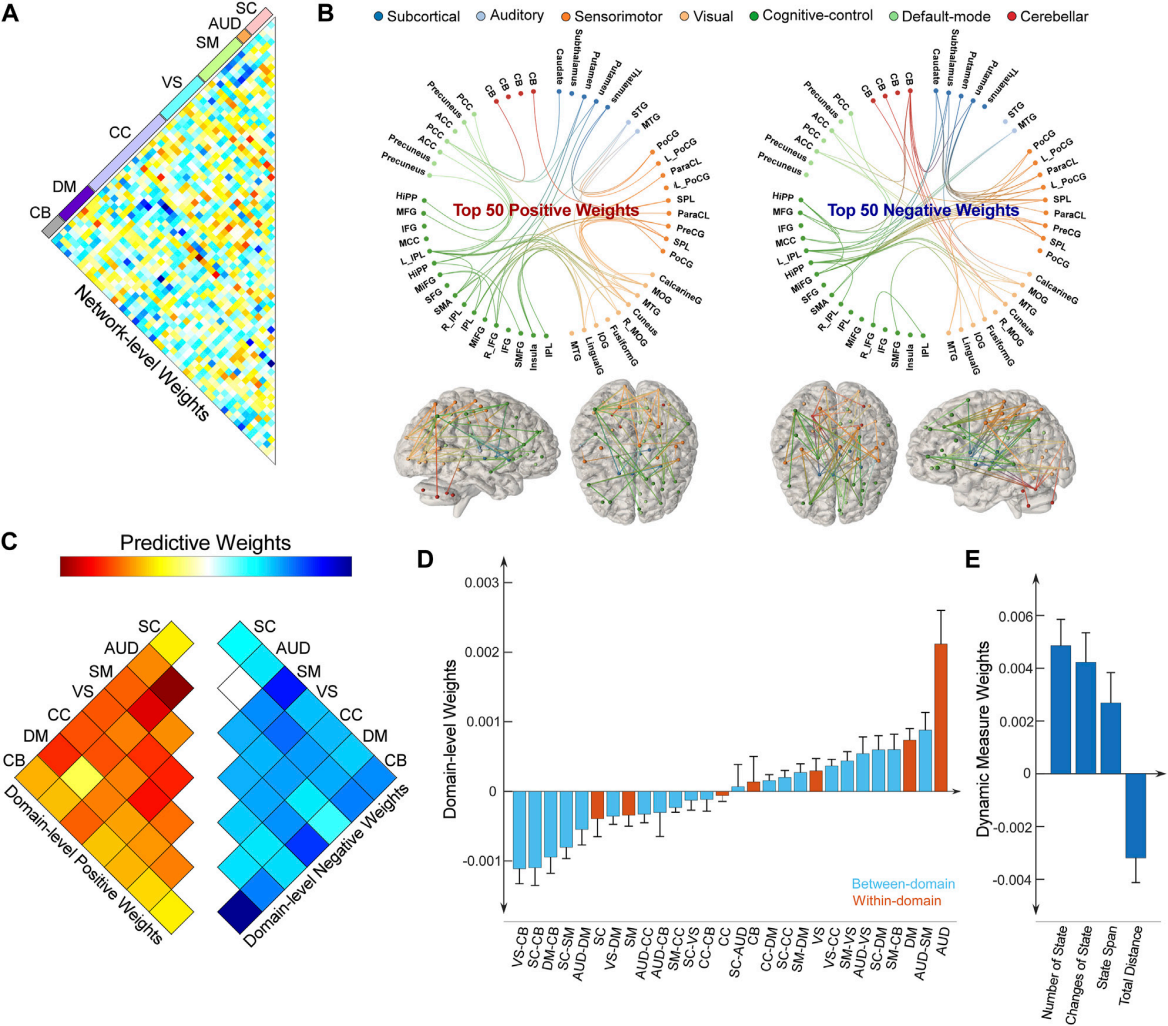
Distributions of weight maps in predicting antidepressant outcomes (HDRS_24_ composite score). **(A)** Distributions of raw predictive weights at the FNC pair level averaged across 10,000 CV rounds. **(B)** Functional connectome plots with the top 50 positive weights and top 50 negative weights. ICNs are arranged into seven functional domains with different colors. **(C)** The cell plots show the domain-level representation of the predictive weights. For each pair of domains (between-domain and within-domain), we averaged predictive weights of all sFNC belonging to that domain pair. Positive weights and negative weights were separately summarized for each domain pair to demonstrate their relative contribution. **(D)** Mean weights distribution of within-domain and between-domain sFNC in the predictive models. Error bars indicate standard deviation. **(E)** Mean weights distribution of dFNC features in the predictive models.

As we leveraged the whole-brain static connectome to make predictions, each sFNC feature obtained a predictive weight representing its contribution to each prediction task. We summarized the weight at the original FNC pair level and at the functional domain level for the predictive model of the HDRS_24_ composite score, with the results displayed in Figure 4. Here we selected the HDRS_24_ for the presentation because the weight maps for antidepressant outcomes are highly similar (vs. HDRS_24_ Depersonalization, *r* = 0.4310, *p* = 1.1 × 10^−63^; vs. HDRS_24_ Anxiety, *r* = 0.5633, *p* = 3.3 × 10^−116^; vs. QIDS Late Sleep, *r* = 0.4800, *p* = 2.5 × 10^−80^). In the prediction of antidepressant outcomes, FNC between SC and SM domains, between SC and CB domains, and between VS and CB domains have prominent negative weights (Figures 4A, B). In contrast, FNC within the AUD domain and DM domain, between AUD and DM domains, and between SM and CB domains show prominent positive weights. CC-related FNC shows different weight patterns. On one hand, FNC between CC and AUD/SM domains demonstrate strongly negative weights, especially for FNC between the middle frontal gyrus and multiple SM ICNs and between MTG and multiple CC ICNs. On the other hand, FNC between CC and DM/VS shows obviously positive weights. Similar patterns are observed when we averaged the weights and displayed the results at the domain-pair level (Figures 4C, D). Regarding the dynamic features, only the total distance of the dynamic range shows negative weight while the other dynamism measures show positive weights.

**FIGURE 5:**
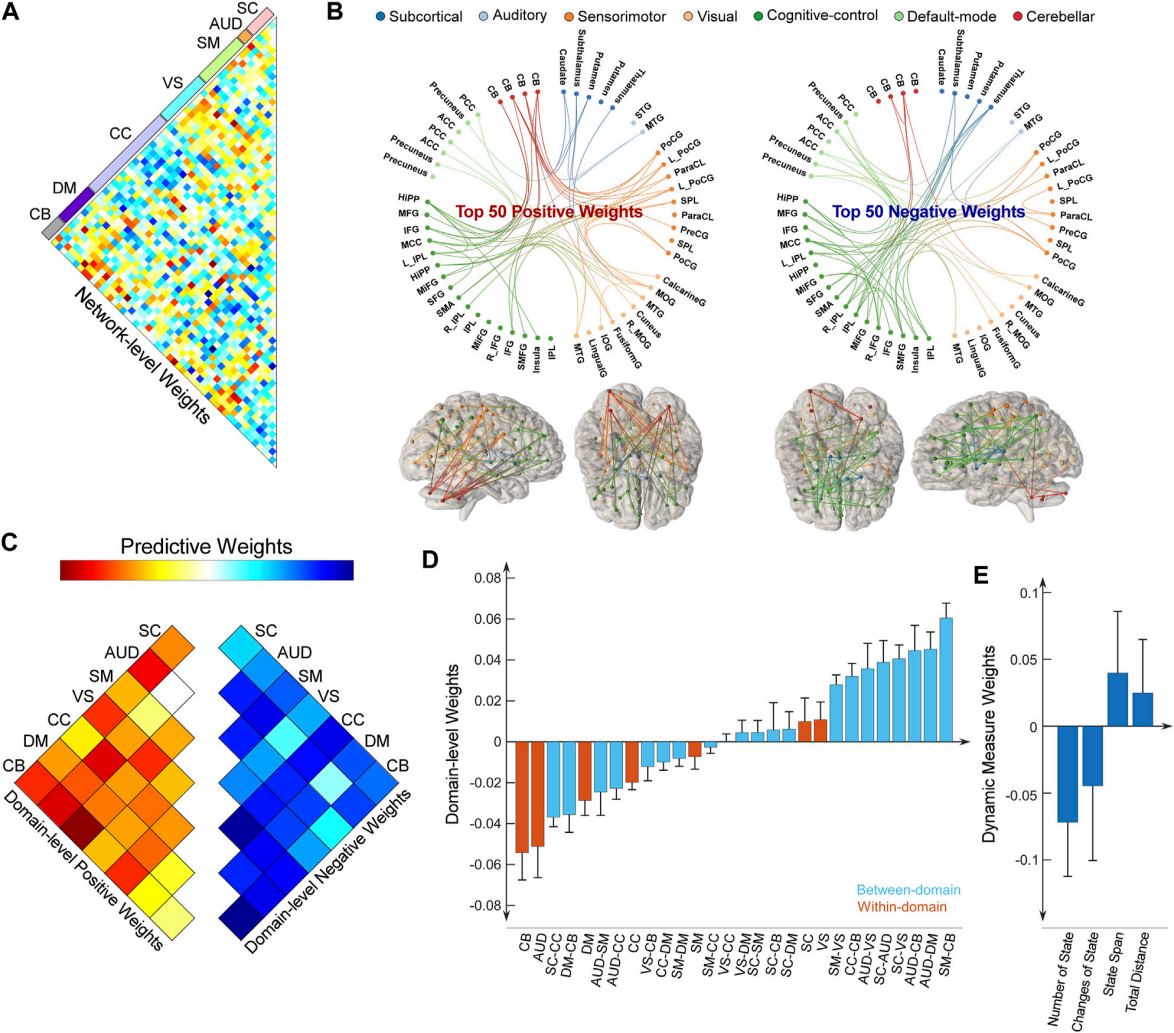
Distributions of weight maps in predicting cognitive changes (HVLT-DR score). **(A)** Distributions of raw predictive weights at the FNC pair level averaged across 10,000 CV rounds. **(B)** Functional connectome plots with the top 50 positive weights and top 50 negative weights. ICNs are arranged into seven functional domains with different colors. **(C)** The cell plots show the domain-level representation of the predictive weights. For each pair of domains (between-domain and within-domain), we averaged predictive weights of all sFNC belonging to that domain pair. Positive weights and negative weights were separately summarized for each domain pair to demonstrate their relative contribution. **(D)** Mean weights distribution of within-domain and between-domain sFNC in the predictive models. Error bars indicate standard deviation. **(E)** Mean weights distribution of dFNC features in the predictive models.

We also summarized the weight for the predictive model of the HVLT-DR score and displayed the results in Figure 5. Note that, the weight maps for the HVLT-DR score and the HVLT-R score are highly correlated (*r* = 0.7576, *p* = 3.6 × 10^−257^). For predicting cognitive changes, FNC within CB, AUD, and DM show prominent negative weights. FNC between SM and CB, and between SM and VS have positive weights (Figures 5A, B). Again, CC-related FNC demonstrates more diverse weight maps. FNC between CC and SC shows negative weights while FNC between CC and VS, and between CC and CB show more positive weights. For the dynamic features, the number of states and the change of states show negative weights while state span and total distance show positive weights. We further examined the overlap of predictive models for antidepressant outcomes and cognitive changes by evaluating the correlation between their weight maps. Their weight maps only show a weakly negative correlation (*r* = −0.0566, *p* = 0.0356). Some functional domains exhibiting great involvement in predicting both antidepressant outcomes and cognitive changes are in the same direction, while some are in opposite directions. For example, FNC between SM and CB shows positive weights for both predictive models of antidepressant outcomes and cognitive changes. In contrast, FNC between SC and CC demonstrate more negative weights in the prediction of cognitive changes while these FNC show more positive weights in the prediction of HDRS changes.

## Discussion

Our work combined a fully automated ICA-based framework with a machine learning model to achieve an individual-level estimation of ECT-induced antidepressant outcomes and cognitive changes. Firstly, the Neuromark is a well-established framework for capturing reliable connectivity features that are comparable across subjects, scans, and sessions (Fu et al., 2020; 2021c; Tu et al., 2020). Unlike atlas-based analysis, this framework allows single-scan variability in network representations, which can therefore retain more scan-specific information in the connectivity features (Du et al., 2020). Secondly, we capitalized on advances in machine learning technology to characterize the connectivity signatures of antidepressant outcomes and cognitive changes in a purely data-driven manner (Jiang et al., 2022). Instead of focusing on isolated FNC pairs, the PLS prediction model concentrates on aggregating thousands of antidepressant outcome- and cognitive change-related FNC changes into a single measure that best captures their neural representations. Moreover, the predictive analysis combined dynamic features with static features in the modeling, which might extend our understanding of ECT outcome prediction dominated by the studies focusing on either connectivity feature alone (Perrin et al., 2012; Redlich et al., 2016; Moreno-Ortega et al., 2019; Dini et al., 2021).

We also envision the potential translational impact of the predictive FC changes for improving personalized ECT treatments. During the ECT evaluation, the clinician will need as much information as possible to individualize anticipated risks and benefits. In acute cases with episodic depressive episodes (e.g., severe depressive episodes associated with poor oral intake and acute suicidality), family history of mood disorders, or relatively short depressive episodes (<2 years), the clinical decision-making will be clear and straight-forward in favor of ECT. In the context of non-episodic depression, extensive treatment failures, or a less severe depression severity, the clinician will benefit from additional predictive biomarkers, such as neuroimaging biomarkers, to facilitate clinical decision-making. Combined with future studies that might build relationships between subjects’ demographic features, pre-ECT imaging, and FC changes, our predictive models will have a significant translational impact, especially in the absence of clear clinical and demographic features.

Existing work has widely established that the depressive brain shows significant relationships with abnormal FC (Greicius et al., 2007; Zeng et al., 2012). Widespread FC abnormalities in major depressive individuals might give rise to a portion of the MDD-related emotional and cognitive disturbances (Zeng et al., 2012). As one of the most popular ECT mechanisms, the neurogenic hypothesis believes that generating new neurons is beneficial for the depressive brain because of its impairment of producing neurons for mood control (Scott et al., 2000; Petrik et al., 2012). Evidence from previous FC studies demonstrates that ECT significantly affects the whole-brain FC, where the resulting FC changes show associations with clinical outcomes (Perrin et al., 2012; Abbott et al., 2014; Wang et al., 2020). A variety of functional networks are involved in ECT, including the frontoparietal control network, the default-mode network (DMN), the subcortical network, and the cerebellum network (Perrin et al., 2012; Wang et al., 2020; Pang et al., 2022). DMN, a system responsible for self-referential information processing, awareness, and memory processing is supposed to play a key role in MDD and is also promising as a target for ECT-induced antidepressant outcomes (Abbott et al., 2013; Mulders et al., 2015; Pang et al., 2022) and cognitive changes (Wang et al., 2020). Our present results are consistent with the previous findings by showing that DMN-related FNC, especially the FNC within DM, significantly contributes to the prediction models of clinical outcomes. Interestingly, we found that DMN-related FNC shows diversely positive and negative effects in the prediction of ECT outcomes, which are in line with previous studies where both increased and decreased DMN FC are observed after ECT (Li et al., 2017; Pang et al., 2022). Our study further extends the previous findings by demonstrating that DMN FC might show reverse effects in predicting antidepressant outcomes and cognitive changes. That is, more decreased FNC within DM is associated with more antidepressant outcomes and fewer cognitive side effects. In addition, our model reveals that the cerebellum is a complicated brain system implicated in ECT responses. Recent studies have shown that the cerebellum is not a pure motor-control system (Schmahmann, 2004; Schmahmann and Caplan, 2006; Schutter and van Honk, 2006) and its abnormalities show prominent associations with neural deficits in brain diseases (Andreasen, 1997; Fatemi et al., 2012; He et al., 2018). The cerebro-cerebellar neuroplasticity may also imply a potential neural pathway for the mitigation of ECT-induced side effects (Depping et al., 2017; Porta-Casteràs et al., 2021; Wei et al., 2021). Based on the cerebellum’s role in cognition and emotional processing (Schmahmann, 2004; Schmahmann and Caplan, 2006; Schutter and van Honk, 2006), we speculate that ECT may impact the cerebellum and modulate its communication with cerebral systems, especially the sensory networks, which therefore influences the allocation of attentional resources to sensory-motor processing, and emotional experience and leads to symptom improvements and cognitive changes (Wang et al., 2018). Collectively, our present work suggests that ECT affects interactions between a variety of functional networks that directly or indirectly impact ECT-induced antidepressant outcomes and cognitive changes.

Most of the aforementioned studies assumed the FC static over the entire resting-state. The assumption of static interactions between brain regions during the resting-state might be limited as it might oversimplify the representation of brain connectivity (Allen et al., 2014). Indeed, fluctuations in FC have long been appreciated in fMRI studies, which might convey neuronally original underpinning of the brain mechanisms (Hutchison et al., 2013). Existing literature has also reported numerous dynamic FC abnormalities in many brain disorders, such as schizophrenia (Damaraju et al., 2014), autism (Fu et al., 2019; Li et al., 2020), depression (Kaiser et al., 2016; Yao et al., 2019), and Parkinson’s disease (Kim et al., 2017). More recently, studies have implemented the dynamic FC analysis to the ECT-related datasets and captured numerous dynamic features which might serve as a potential biomarker of the ECT outcomes (Fu et al., 2021c; Dini et al., 2021; Liu et al., 2022). Dynamic FC might convey diseases- or treatments-related mechanisms that cannot be identified by static analysis (Hutchison et al., 2013; Matsui et al., 2019). However, previous work usually focused on either static or dynamic FC separately, failing to answer whether dynamic FC can provide additional information to its static counterpart. The apparent associations between ECT responses and both static and dynamic FC is a basic motivation that drives the current study to investigate the nature of their relationship by incorporating them into the predictive models of ECT outcomes. For the first time, our study demonstrates that the global dynamism of whole-brain FC provides complementary information to the static FC, leading to an improved prediction accuracy of antidepressant outcomes. The changes in global dynamism might reflect how ECT influences the brain dynamic range and fluidity, providing additional information on the ECT-related neuroplasticity of the brain. One potential explanation is that ECT changes the brain dynamism to normalize the weakness in brain circuits related to cognitive control and depressive deficits in executive functioning (MacDonald et al., 2000; Kaiser et al., 2016). Another interesting finding is that brain dynamism does not provide additional information to the static FC in the prediction of cognitive changes. We argue that this may be partially due to the inherently heterogeneous ECT impact on cognitive functions (Stern et al., 1994; Stern, 2002). Specifically, individuals with higher premorbid intelligence can better compensate for the impact of ECT on cognitive functions (Stern et al., 1994; Stern, 2002). The brain dynamism might not be sensitive to the individual difference in intelligence and therefore fails to predict the heterogeneous changes in cognition.

Our study should be considered in light of some potential limitations. First, our study is based on an ECT dataset where the participants received ECT series with different amplitudes. Previous studies have found that the electric field determined by the amplitude shows significant associations with brain neuroplasticity, antidepressant outcomes, and cognitive side effects (Argyelan et al., 2019; Deng et al., 2021). Although we have replicated the majority of the findings in the main text by regressing the confounding effect of amplitude in the Supplementary Material, the neuroimaging signatures might have different representations in different amplitudes. In future studies with more samples collected with each pulse amplitude, we can validate and extend our prediction analysis to each amplitude respectively. Second, the prediction models of antidepressant outcome and cognitive change were developed based on linear models, which might ignore the potential non-linear relationships between FC and ECT outcomes. Third, our present work only considered static and dynamic FC features in the prediction of clinical outcomes. However, ECT-induced structural changes have also been widely associated with clinical outcomes (Argyelan et al., 2019; Deng et al., 2021). It is still an open question whether structural and functional neuroplasticity plays overlapping or complementary roles in antidepression-related circuitry. Investigations incorporating multi-modal imaging features can help to elucidate the relationships between structural and functional neuroplasticity with ECT and might provide better prediction accuracy of ECT response. Fourth, the present work built the predictive model only for ECT-induced memory changes. We mainly focused on the memory domain because it is the most impacted cognitive domain associated with ECT (Semkovska and McLoughlin, 2010). Our prediction models can be extended to the prediction of other cognitive domains to draw a more comprehensive connectivity-based signature of cognitive impairments associated with ECT. Fifth, the prediction model was not built using the dynamic features alone because features with such a small dimension are not suitable for the PLSR method. In future studies, we can investigate other dynamic features with larger dimensions and examine their predictive abilities for ECT outcomes directly. Finally, it is also of interest whether and how pre-ECT imaging and previous antidepressant exposure influence the predictive models. Incorporating antidepressant type, dose, and duration into pre-ECT prediction modeling is a promising future direction that will increase the generalizability and translational impact of predictive modeling. Unfortunately, our current dataset is not sufficiently powered for this type of analysis. We have initiated a multi-site ECT-imaging investigation that will capture pre-treatment medications and a number of failed antidepressant trials. When completed, this large dataset (*n* > 200) will allow us to incorporate antidepressant classes and pre-ECT clustered FC patterns into the predictive modeling approach for both antidepressant and cognitive outcomes.

## Conclusion

In this study, we investigated the extent to which ECT-induced antidepressant outcomes and cognitive changes overlap in brain connectivity *via* a technique from multivariate predictive modeling. Our findings first demonstrate that changes in static connectivity can predict ECT-induced clinical outcomes. Although sharing a few common patterns, antidepressant outcomes and cognitive changes have specific neural representations in brain connectivity. More importantly, we found that adding dynamic connectivity information to static connectivity can significantly enhance the performance of predicting antidepressant outcomes only (not for memory changes). It is widely believed that connectivity dynamics evaluate brain interactions at a finer time scale and thus might provide more information to the static analysis. However, our findings indicate that dynamic connectivity does not always provide additional neural signatures to its static counterpart, which might depend on the specific task to be investigated. In sum, our work offers new insights into the complex relationships between static and dynamic brain connectivity in ECT outcome prediction. Tracking the patterns of static and dynamic connectivity changes may better characterize the antidepressant efficacy and guide people to provide an optimal ECT paradigm to reverse the symptoms in depressive patients.

## Data Availability

The datasets presented in this study can be found in online repositories. The names of the repository/repositories and accession number(s) can be found below: https://nda.nih.gov/. The codes of the Neuromark framework have been released and integrated into the group ICA Toolbox (https://trendscenter.org/software/gift/), which can be downloaded and used directly by users worldwide. Other MATLAB codes of this study can be obtained from the corresponding authors.

## Abbreviations

AUD, auditory domain; CB, cerebellum domain; CC, cognitive-control domain; CV, cross-validation; dFNC, dynamic functional network connectivity; DM, default-mode domain; DMN, default-mode network; ECT, electroconvulsive therapy; fMRI, functional magnetic resonance imaging; FC, functional connectivity; HDRS24, Hamilton Depression Rating Scale 24-item; HVLT, Hopkins Verbal Learning Test; ICA, independent component analysis; ICN, intrinsic connectivity network; MDD, major depressive disorder; MNI, Montreal Neurological Institute; PLSR, partial least square regression; QC, quality control; QIDS, Quick Inventory of Depressive Symptomatology; sFNC, static functional network connectivity; SC, subcortical domain; SM, sensorimotor domain; TC, time-course; TR, repetition time; VS, visual domain.
