# Supplementary material for: Predictive signature of static and dynamic functional connectivity for ECT clinical outcomes
Source: Front Pharmacol. 2023 Jan 23;14:1102413. doi: 10.3389/fphar.2023.1102413 (PMC9899999; doi:10.3389/fphar.2023.1102413)
Supplement: Supplementary file 1 [file Table1.DOCX]

Supplementary Materials for

“Predictive Signature of Static and Dynamic Functional Connectivity for ECT Clinical Outcomes”

# Demographics and Clinical Assessments.

A detailed description of the study design and clinical assessments can be found in (Abbott et al., 2021) (ClinicalTrials.gov Identifier: NCT02999269). Subjects aged between 50 to 80 years, with a diagnosis of major depressive disorder and meeting the clinical indication for Electroconvulsive therapy (ECT) were recruited in this study. We excluded subjects if they have 1) neurological or neurodegenerative disorder, 2) psychiatric conditions, 3) substance (except nicotine) or alcohol use disorder, or 4) contraindications to magnetic resonance imaging (MRI). In total 62 subjects participated in this study. 50 participants passed the Neuromark quality control (QC).

At each visit, trained raters performed comprehensive clinical assessments for each participant. For all the visits (v1, v2, and v3), the Hamilton Depression Rating Scale 24-item (HDRS_24_) and Hopkins Verbal Learning Test (HVLT)-Revised scores were collected to measure the primary clinical and cognitive outcomes. The HVLT measured learning and immediate recall of 12 semantically related words across three trials. In this study, we used Forms 1 and 4 of HVLT and randomized the order across participants to minimize the practice effects.

Demographic information of the participants passing the Neuromark QC is provided in Table S1.

Demographics and clinical characteristics of participants

|  | Characteristics -- MDD (n=50) | |  | T statistic |
| --- | --- | --- | --- | --- |
|  | Last Visit | v1 | v3 | p value |
| Age (years) | 65.54 ± 8.92 | n/a | n/a | n/a |
| Gender (female/male) | 35/15 | n/a | n/a | n/a |
| treatment arms (600/700/800 mA) | 9/12/29 | 14/18/18 | 9/12/29 | n/a |
| ECT treatments | 11 ± 3.25 | n/a | n/a | n/a |
| Pulse width (0.3/1 ms) | 21/29 | 38/12 | 21/29 | n/a |
| HDRS | 14.88 ± 10.47 | 36.22 ± 7.03 | 14.88 ± 10.47 | 8.50 × 10^-18^ |
| QIDS | 0.92 ± 1.02 | 1.72 ± 1.14 | 0.92 ± 1.02 | 0.0014 |
| HVLT-R | 35.10 ± 13.81 | 36.66 ± 15.64 | 35.10 ± 13.81 | 0.5168 |
| HVLT-DR | 30.53 ± 9.88 | 31.62 ± 11.01 | 30.53 ± 9.88 | 0.4451 |

ECT = Electroconvulsive Therapy; HDRS = Hamilton Depression Rating Scale; HVLT = Hopkins Verbal Learning Test; MDD = Major Depressive Disorder; QIDS = Quick Inventory of Depressive Symptomatology.

# Image Preprocessing

The resting-state fMRI data were preprocessed using the FMRIB Software Library v6.0 (FSL) toolbox and Statistical Parametric Mapping 12 (SPM 12) toolbox. The FSL tool *topup* (Andersson et al., 2003; Smith et al., 2004) was used to estimate the susceptibility-induced off-resonance field based on the volumes acquired with phase encoding in the anterior-posterior (AP) direction and volumes with phase encoding in the posterior-anterior (PA) direction. The estimated field maps were then used to correct the distortion in the fMRI data using *applytopup*. 10 initial scans were discarded followed by the distortion correction. We further performed a slice-timing correction to account for the timing difference in slice acquisition and then a rigid body motion correction to correct the head motion. Finally, the fMRI data were subsequently warped into the standard Montreal Neurological Institute (MNI) space using an echo-planar imaging (EPI) template (resampled to 3 × 3 ×3 mm^3^ isotropic voxels) and were smoothed using a Gaussian kernel with a full width at half maximum (FWHM) = 6 mm.

# Neuromark Quality Control

Firstly, we excluded subjects if their head motions were larger than 3 mm translations or 3° rotations. Previous studies have shown that fMRI data with large head motions might influence functional connectivity.

Secondly, we examined whether the subjects’ scans have good normalization to the EPI template. fMRI data with good normalization to the EPI template is important for the independent component analysis (ICA). Here, we compared the individual mask with the group mask to ensure that we have a high-quality mask and the fMRI data for further analysis. The effectiveness of this method has been proven in previous studies (Fu et al., 2020, 2021; Du et al., 2021; Li et al., 2021).

Specifically, we calculated the individual mask using the first volume of each scan by setting voxels that are greater than 90% of the whole brain mean to 1. Then we computed a group mask by setting voxels to 1 if they were included in more than 90% of the scans. Once we had the group mask and the individual masks, we calculated the spatial correlations between them. The spatial correlations were calculated using voxels within the top 10 slices of the mask, within the bottom 10 slices of the mask, and within the whole mask, resulting in three correlation values for each scan. If a scan has a top-10-slices correlation larger than 0.75, a bottom-10-slices correlation larger than 0.55, and a whole-brain correlation larger than 0.8, we considered this scan having good normalization to the standard space. Subjects with at least one good scan (passed the Neuromark QC) in both v1 and v3 sessions were used in the further analysis.

# Neuromark Framework

The Neuromarkr framework used two large healthy controls datasets, the human connectome project (HCP, 823 subjects after the subject selection) and the genomics superstruct project (GSP, 1005 subjects after the subject selection) to construct a set of reproducible network templates, which were used as the reference to estimate single-scan ICNs and the corresponding time courses (TCs) for the ECT dataset.

For the construction of network templates, a group ICA with a model order of 100 was performed on the GSP and HCP datasets respectively. The independent components (ICs) estimated for each dataset were then matched by evaluating the correlation between their group-level spatial maps. Previous studies have shown that a correlation ≥ 0.25 represents a significant correspondence (p < 0.005, corrected) between components (Smith et al., 2009). Here in the Neuromark framework, we considered a component consistent and reproducible across HCP and GSP if it has a matched component with spatial correlation ≥ 0.4. We used a higher threshold because we would like to identify more reliable and consistent ICs. Five experts evaluated the reproducible IC pairs by examining their peak activations and low-frequency fluctuations of TCs. 53 pairs of ICs were identified as intrinsic connectivity networks (ICNs), arranged into seven functional domains. The less noisy ICN templates from the GSP dataset (Note that there are 53 ICNs from HCP which have similar spatial patterns) were finally set as the reference to estimate the components and TCs for each scan of the ECT data. Details of the ICNs labeling, coordinates, and spatial maps are provided in Table S2 and Figure S1.

# Neuromark Intrinsic Connectivity Networks (ICNs)

Labels and Peak Coordinates for ICNs

| **ICNs** | **X** | **Y** | **Z** |
| --- | --- | --- | --- |
| **Sub-cortical domain (SC)** | | | |
| Caudate (69) | 6.5 | 10.5 | 5.5 |
| Subthalamus/hypothalamus (53) | -2.5 | -13.5 | -1.5 |
| Putamen (98) | -26.5 | 1.5 | -0.5 |
| Caudate (99) | 21.5 | 10.5 | -3.5 |
| Thalamus (45) | -12.5 | -18.5 | 11.5 |
| **Auditory domain (AUD)** | | | |
| Superior temporal gyrus ([STG], 21) | 62.5 | -22.5 | 7.5 |
| Middle temporal gyrus ([MTG], 56) | -42.5 | -6.5 | 10.5 |
| **Sensorimotor domain (SM)** | | | |
| Postcentral gyrus ([PoCG], 3) | 56.5 | -4.5 | 28.5 |
| Left postcentral gyrus ([L PoCG], 9) | -38.5 | -22.5 | 56.5 |
| Paracentral lobule ([ParaCL], 2) | 0.5 | -22.5 | 65.5 |
| Right postcentral gyrus ([R PoCG], 11) | 38.5 | -19.5 | 55.5 |
| Superior parietal lobule ([SPL], 27) | -18.5 | -43.5 | 65.5 |
| Paracentral lobule ([ParaCL], 54) | -18.5 | -9.5 | 56.5 |
| Precentral gyrus ([PreCG], 66) | -42.5 | -7.5 | 46.5 |
| Superior parietal lobule ([SPL], 80) | 20.5 | -63.5 | 58.5 |
| Postcentral gyrus ([PoCG], 72) | -47.5 | -27.5 | 43.5 |
| **Visual domain (VS)** | | | |
| Calcarine gyrus ([CalcarineG], 16) | -12.5 | -66.5 | 8.5 |
| Middle occipital gyrus ([MOG], 5) | -23.5 | -93.5 | -0.5 |
| Middle temporal gyrus ([MTG], 62) | 48.5 | -60.5 | 10.5 |
| Cuneus (15) | 15.5 | -91.5 | 22.5 |
| Right middle occipital gyrus ([R MOG], 12) | 38.5 | -73.5 | 6.5 |
| Fusiform gyrus (93) | 29.5 | -42.5 | -12.5 |
| Inferior occipital gyrus ([IOG], 20) | -36.5 | -76.5 | -4.5 |
| Lingual gyrus ([LingualG], 8) | -8.5 | -81.5 | -4.5 |
| Middle temporal gyrus ([MTG], 77) | -44.5 | -57.5 | -7.5 |
| **Cognitive-control domain (CC)** | | | |
| Inferior parietal lobule ([IPL], 68) | 45.5 | -61.5 | 43.5 |
| Insula (33) | -30.5 | 22.5 | -3.5 |
| Superior medial frontal gyrus ([SMFG], 43) | -0.5 | 50.5 | 29.5 |
| Inferior frontal gyrus ([IFG], 70) | -48.5 | 34.5 | -0.5 |
| Right inferior frontal gyrus ([R IFG], 61) | 53.5 | 22.5 | 13.5 |
| Middle frontal gyrus ([MiFG], 55) | -41.5 | 19.5 | 26.5 |
| Inferior parietal lobule ([IPL], 63) | -53.5 | -49.5 | 43.5 |
| Left inferior parietal lobue ([R IPL], 79) | 44.5 | -34.5 | 46.5 |
| Supplementary motor area ([SMA], 84) | -6.5 | 13.5 | 64.5 |
| Superior frontal gyrus ([SFG], 96) | -24.5 | 26.5 | 49.5 |
| Middle frontal gyrus ([MiFG], 88) | 30.5 | 41.5 | 28.5 |
| Hippocampus ([HiPP], 48) | 23.5 | -9.5 | -16.5 |
| Left inferior parietal lobue ([L IPL], 81) | 47.5 | 5.5 | 22.5 |
| Middle cingulate cortex ([MCC], 37) | -15.5 | 20.5 | 37.5 |
| Inferior frontal gyrus ([IFG], 67) | 39.5 | 44.5 | -0.5 |
| Middle frontal gyrus ([MiFG], 38) | -26.5 | 47.5 | 5.5 |
| Hippocampus ([HiPP], 83) | -24.5 | -36.5 | 1.5 |
| **Default-mode domain (DM)** | | | |
| Precuneus (32) | -8.5 | -66.5 | 35.5 |
| Precuneus (40) | -12.5 | -54.5 | 14.5 |
| Anterior cingulate cortex ([ACC], 23) | -2.5 | 35.5 | 2.5 |
| Posterior cingulate cortex ([PCC], 71) | -5.5 | -28.5 | 26.5 |
| Anterior cingulate cortex ([ACC], 17) | -9.5 | 46.5 | -10.5 |
| Precuneus (51) | -0.5 | -48.5 | 49.5 |
| Posterior cingulate cortex ([PCC], 94) | -2.5 | 54.5 | 31.5 |
| **Cerebellar domain (CB)** | | | |
| Cerebellum ([CB], 13) | -30.5 | -54.5 | -42.5 |
| Cerebellum ([CB], 18) | -32.5 | -79.5 | -37.5 |
| Cerebellum ([CB], 4) | 20.5 | -48.5 | -40.5 |
| Cerebellum ([CB], 7) | 30.5 | -63.5 | -40.5 |


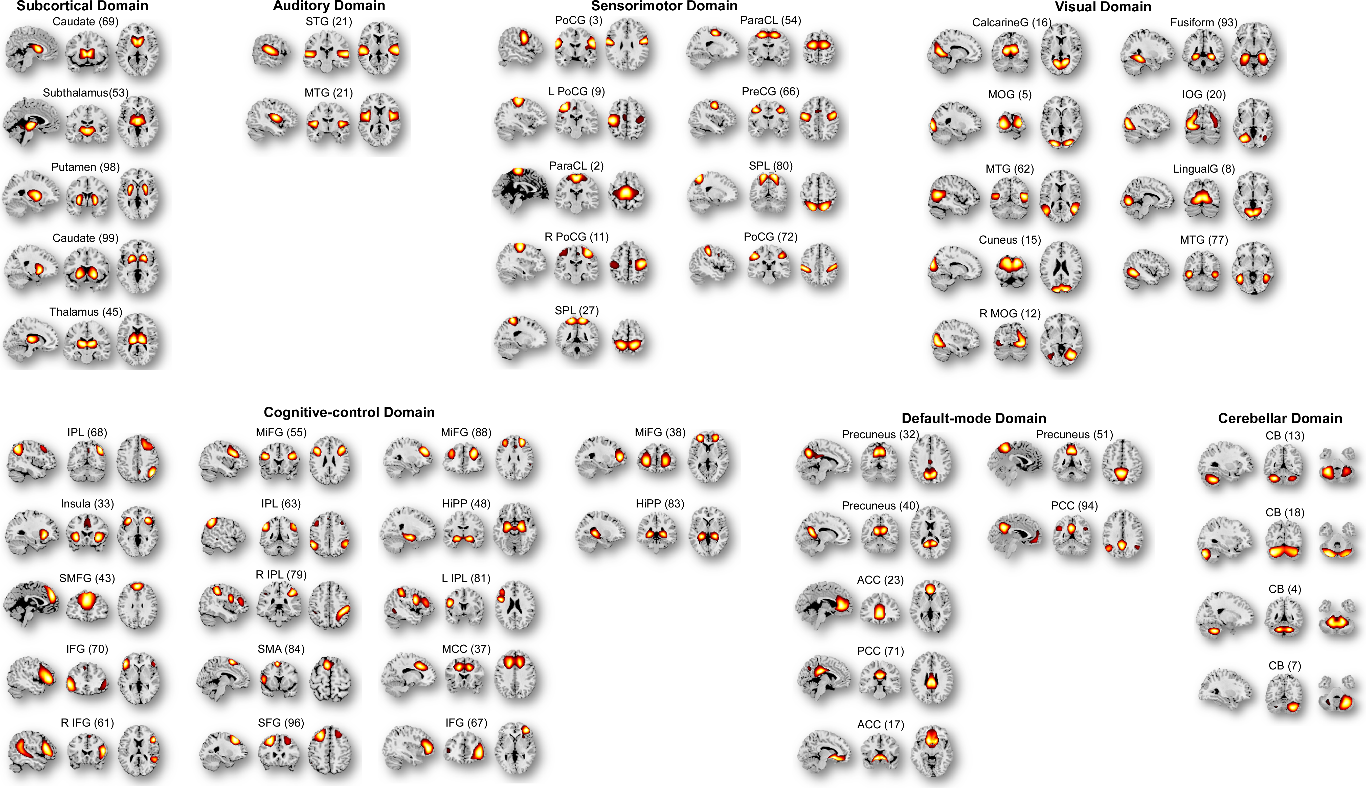


**Spatial maps of 53 selected ICNs.** 53 components are shown and thresholded at |*t*|>10, where one-sample t-statistics have been computed across the single-subject spatial maps. Sagittal, coronal, and axial slices are shown at the maximal t-statistic for clusters larger than 3 cm^3^. The ICNs are arranged into 7 functional domains according to their anatomical and functional prior knowledge.

# Replication of Connectome-Based Prediction by Regressing Out Confounding Effects

To demonstrate that the connectome-based prediction is not biased by the confounding effects, we further built predictive models for symptom scores and cognitive performance using the cleaned static functional network connectivity (sFNC) and dynamic FNC (dFNC) features. Specifically, before building the prediction model, a general linear model (GLM) was used to regress out the potential confounding effects from both sFNC and dFNC features (changes between v1 and v3). For each feature, the effects of age, gender, pulse amplitude, ECT treatment, and pulse width were regressed out if they have significant associations with this feature (p < 0.05). Then the cleaned features were used in the PLSR model for predicting changes in the symptom scores and cognitive performance. We performed the same prediction framework as introduced in the main text.

The prediction results are highly consistent with the findings presented in the main text, although the overall accuracy slightly dropped. The sFNC features can successfully predict the HDRS_24_ composite score (r = 0.4544 ± 0.0512, R^2^ = 0.2091 ± 0.0457, permutation test p < 2.0 × 10^-3^). When adding dynamic features to the prediction model, the combined features can achieve better prediction accuracy (r = 0.4901 ± 0.0441, R^2^ = 0.2421 ± 0.0426, permutation test p < 4.0 × 10^-3^). The two-sample t-test shows that using combined features provides higher prediction accuracy compared to using only static features (t = 16.6977, p = 1.1 × 10^-58^). Similarly. combined static features with dynamic features can increase the prediction accuracy of depersonalization score and anxiety score (r = 0.4351 ± 0.0548, t = 8.0001, p = 2.1 × 10^-15^, and r = 0.4509 ± 0.0445, t = 57.5760, p = 1.0 × 10^-30^). The prediction results for the cognitive performance show that sFNC features can predict the HVLT-DR score and HVLT-R score (r = 0.5005 ± 0.0382, R^2^ = 0.2520 ± 0.0379, and r = 0.4288 ± 0.0426, R^2^ = 0.1857 ± 0.0374, permutation test p < 5.0 × 10^-3^). Consistent with the results in the main text, adding dFNC features to the model did not improve the prediction accuracy (t = -23.7877, p = 2.5 × 10^-30^, and t = -27.4125, p = 1.1 × 10^-30^).

Prediction Accuracy for Symptom and Cognition Changes

| **Changes of clinical outcomes** | **sFNC only** | **sFNC + dFNC** | **T value** | **P value** |
| --- | --- | --- | --- | --- |
| **HDRS_24_** | 0.4544 ± 0.0512 | 0.4901 ± 0.0441 | 16.6977 | 1.1 × 10^-58^ |
| **HDRS_24_ depersonalization** | 0.4148 ± 0.0583 | 0.4351 ± 0.0548 | 8.0001 | 2.1 × 10^-15^ |
| **HDRS_24_ anxiety** | 0.3223 ± 0.0548 | 0.4509 ± 0.0445 | 57.5760 | 1.0 × 10^-30^ |
| **QIDS late sleep** | 0.4176 ± 0.0521 | 0.4584 ± 0.0470 | 18.4082 | 5.0 × 10^-30^ |
| **HVLT-DR** | 0.5005 ± 0.0382 | 0.4587 ± 0.0405 | -23.7877 | 2.5 × 10^-30^ |
| **HVLT-R** | 0.4288 ± 0.0426 | 0.3729 ± 0.0485 | -27.4125 | 1.1 × 10^-30^ |

# References

Abbott, C. C., Quinn, D., Miller, J., Ye, E., Iqbal, S., Lloyd, M., et al. (2021). Electroconvulsive Therapy Pulse Amplitude and Clinical Outcomes. *Am. J. Geriatr. Psychiatry* 29, 166–178. doi:10.1016/j.jagp.2020.06.008.

Andersson, J. L. R., Skare, S., and Ashburner, J. (2003). How to correct susceptibility distortions in spin-echo echo-planar images: Application to diffusion tensor imaging. *Neuroimage* 20, 870–888. doi:10.1016/S1053-8119(03)00336-7.

Du, Y., Fu, Z., Xing, Y., Lin, D., Pearlson, G., Kochunov, P., et al. (2021). Evidence of shared and distinct functional and structural brain signatures in schizophrenia and autism spectrum disorder. *Commun. Biol.* 4, 1–16. doi:10.1038/s42003-021-02592-2.

Fu, Z., Sui, J., Espinoza, R., Narr, K., Qi, S., Sendi, M. S. E., et al. (2021). Whole-brain Functional Connectivity Dynamics associated with Electroconvulsive Therapy Treatment Response. *Biol. Psychiatry Cogn. Neurosci. Neuroimaging*. doi:10.1016/j.bpsc.2021.07.004.

Fu, Z., Sui, J., Turner, J. A., Du, Y., Assaf, M., Pearlson, G. D., et al. (2020). Dynamic functional network reconfiguration underlying the pathophysiology of schizophrenia and autism spectrum disorder. *Hum. Brain Mapp.*, hbm.25205. doi:10.1002/hbm.25205.

Li, K., Fu, Z., Luo, X., Zeng, Q., Huang, P., Zhang, M., et al. (2021). The Influence of Cerebral Small Vessel Disease on Static and Dynamic Functional Network Connectivity in Subjects along Alzheimer’s Disease Continuum. *Brain Connect.* 11, 189–200. doi:10.1089/brain.2020.0819.

Smith, S. M., Fox, P. T., Miller, K. L., Glahn, D. C., Fox, P. M., Mackay, C. E., et al. (2009). Correspondence of the brain’s functional architecture during activation and rest. *Proc. Natl. Acad. Sci. U. S. A.* 106, 13040–13045. doi:10.1073/pnas.0905267106.

Smith, S. M., Jenkinson, M., Woolrich, M. W., Beckmann, C. F., Behrens, T. E. J., Johansen-Berg, H., et al. (2004). Advances in functional and structural MR image analysis and implementation as FSL. *Neuroimage* 23, S208–S219. doi:10.1016/j.neuroimage.2004.07.051.
